# Supplementary material for: Usefulness of 18F-FDG PET-CT in the Management of Febrile Neutropenia: A Retrospective Cohort from a Tertiary University Hospital and a Systematic Review
Source: Microorganisms. 2024 Jan 31;12(2):307. doi: 10.3390/microorganisms12020307 (PMC10893204; doi:10.3390/microorganisms12020307)
Supplement: Supplementary file 1 [file microorganisms-12-00307-s001.zip › S1.ANTI-INFECTIVE PROPHYLAXIS PROTOCOL.pdf]

SUPPLEMENTARY MATERIAL. ANTI-INFECTIVE PROPHYLAXIS PROTOCOL.

Prophylaxis in hematopoietic stem cell transplantation

Definitions:
- HSCT with standard infectious risk: Autologous transplant of hemopoietic progenitors from peripheral blood without manipulation of the inoculum (depletion of T lymphocytes).
- HSCT with high infectious risk: Autologous transplants of peripheral blood hemopoietic progenitors with manipulation of the inoculum (depletion of T lymphocytes), and all allogeneic transplants. Within this group, haploidentical transplants, with manipulation of the inoculum (depletion of T lymphocytes) and dual transplants will be considered to have a very high infectious risk.

1. Antibacterial prophylaxis

⇨ Levofloxacin 500 mg/24 h PO from admission.

- Standard risk: until reaching PMN> 0.5 x109/L.
- High risk: Maintain until day +60
- If intolerance to quinolones, consider introducing a 3rd generation Cephalosporin.
- If empiric antibiotic therapy is required for the treatment of febrile neutropenia in patients at standard infectious risk, temporarily suspend it.

2. Prophylaxis against HSV and HZV

⇨ Acyclovir:

- Standard risk: After admission (and at least 1 day before starting conditioning) start acyclovir 500 mg/12h IV. Once PNN > 0.5x 109/l is reached, modify it with acyclovir 800mg/24h PO. It should be maintained for at least 6-12 months.
- High risk: After admission (and at least 1 day before starting conditioning), start acyclovir 500 mg/8h IV until oral tolerance, then 800 mg/12h PO. It should be maintained for at least one year after the transplant. It is recommended to maintain prophylaxis in patients who continue with immunosuppressive treatment or if GVHD on steroid treatment.

⇨ Alternatively, use Valacyclovir 500mg/12h PO.

3. Prophylaxis against CMV

- Active surveillance of CMV replication is recommended by performing plasma PCR twice a week in patients at high infectious risk.
- If reactivation occurs, preemptive treatment will be performed.

4. Prophylaxis against P. jirovecii

⇨ Cotrimoxazole: 800 mg Trim/160 mg Sulf IV/12h, from day –7 to –2 (both inclusive). Restart when PMN >1.00 x109/l (and remain stable) at a dose of Sulfomethoxazole/trimethoprim 800/160mg every 12h PO 2-3 days a week

- Standard risk: maintain for 3-6 months after transplant.
- High risk: maintain for at least 6 months, while immunosuppressive treatment is maintained or as long as chronic GVHD lasts. Consider administration of nebulized pentamidine on day -1.

5. Antifungal prophylaxis

⇨ Fluconazole 400 mg/24 h IV or PO from admission and until PMN >0.5 x109/l in patients with autologous transplant (standard infectious risk).

⇨ In patients with high infectious risk transplants:

- Of choice, posaconazole 300 mg/24h IV or PO depending on oral tolerance. It should be maintained during the period of neutropenia, until at least 90 days after the transplant or until the GVHD resolves.
  Other options:
  - Vorizonazole 200mg/12h PO
  - Micafungin 50mg/24h IV.
  - Amphotricin B liposomal 5mg/kg IV 2 times a week.

- In patients with a previous history of treated fungal infection, from the onset of neutropenia (PNN <0.5 x109/l) secondary prophylaxis will be initiated with the most appropriate antifungal treatment at the clinician's discretion.

6. Prophylaxis against Toxoplasma.

⇨ In patients with very high infectious risk transplants (cord and haploidentical), Azithromycin 1g 2 times/week IV or PO until engraftment (then replace with trimethoprim-sulfomethoxazole 160/800mg every 24h orally 3 times a week, OF CHOICE). It should be maintained for a period of at least 6 months, at least as long as the patient remains immunosuppressed.

⇨ In case of intolerance to trimethoprim-sulfomethoxazole (rash, gastrointestinal intolerance), atovaquone 1500 mg/day PO is recommended as an alternative.

7. Prophylaxis against BK virus

- It will be performed in patients with high infectious risk. Prophylaxis of hemorrhagic cystitis caused by BK virus will be carried out through hyperhydration and bladder lavage in order to reduce urothelial damage, especially when myeloablative regimens based on the use of cyclophosphamide, busulfan or total body irradiation are used.
- Prophylaxis with levofloxacin will be carried out following the instructions in the “antibacterial prophylaxis” section.

8. Prophylaxis against M. tuberculosis.

⇨ If Mantoux + pre-BMT or history of tuberculosis disease: Isoniazid (Cemidón→) 300 mg (2 tablets) PO at breakfast for 6 months.

9. Prophylaxis against hepatotrophic viruses.

- HBV: To prevent reactivation, antiviral prophylaxis (entecavir, tenofovir, lamivudine) is recommended for at least 6-12 months after completing treatment.

- HCV: Consider concomitant treatment with anti-HCV treatment according to Digestive System criteria. It is recommended to monitor viral load and liver enzymes monthly or according to clinical criteria.

- HIV: retroviral treatment according to infectious disease criteria. Consider drug interactions.

10. In all patients, vaccination program according to the vaccination schedule
