# Supplementary material for: Usefulness of 18F-FDG PET-CT in the Management of Febrile Neutropenia: A Retrospective Cohort from a Tertiary University Hospital and a Systematic Review
Source: Microorganisms. 2024 Jan 31;12(2):307. doi: 10.3390/microorganisms12020307 (PMC10893204; doi:10.3390/microorganisms12020307)
Supplement: Supplementary file 1 [file microorganisms-12-00307-s001.zip › S2.Search Strategy.pdf]

SEARCH STRATEGY

PUBMED STRATEGY

#1	Tomography, Emission-Computed [Mesh]	133.376
#2	Neutropenia [Mesh]	20.523
#3	#1 AND #2	44
#4	“neutropenia fever”[title/Abstract:~2]	1.390
#5	Febrile neutropenia OR neutropenic fever	11.941
#6	#4 OR #5	12.692
#7	18F-FDG PET/CT OR FDG-PET/CT OR PET SCAN OR positron emission tomograpy OR PET SCANS OR PET imaging OR PET-CT scan OR PET-CT scans OR PET CT scan OR PET CT Scans OR positro emission tomography-computed tomography OR tomography emission computed OR computed emission scintigraphy OR computerized emission Tomography OR ct scan radionuclide	182.959
#8	#6 AND #7	93
#9	#6 OR #8	119

EMBASE STRATEGY

#1	**'18f-fdg pet/ct'**:ti,ab OR **'fdg-pet/ct'**:ti,ab OR **'pet scan'**:ti,ab OR **'positron emission tomograpy'**:ti,ab OR **'pet scans'**:ti,ab OR **'pet imaging'**:ti,ab OR **'pet-ct scan'**:ti,ab OR **'pet-ct scans'**:ti,ab OR **'pet ct scan'**:ti,ab OR **'pet ct scans'**:ti,ab OR **'positro emission tomography-computed tomography'**:ti,ab OR **'tomography emission computed'**:ti,ab OR **'computed emission scintigraphy'**:ti,ab OR **'computerized emission tomography'**:ti,ab OR **'ct scan radionuclide'**:ti,ab	83.476
#2	(neutropenia NEAR/2 fever):ti,ab OR “febrile neutropenia”:ti,ab OR “neutropenic fever”:ti,ab	22.380
#3	#1 AND #2	176

COCHRANE STRATEGY

#1	“Positron Emission Tomography Computed Tomography” [Mesh]	2552
#2	“Febrile neutropenia” [Mesh]	5115
#3	#1 AND #2	46
